# Decoding Microbial Plastic Colonisation: Multi‐Omic Insights Into the Fast‐Evolving Dynamics of Early‐Stage Biofilms

**DOI:** 10.1002/pmic.202400208

**Published:** 2025-01-06

**Authors:** Charlotte E. Lee, Lauren F. Messer, Ruddy Wattiez, Sabine Matallana‐Surget

**Affiliations:** ^1^ Division of Biological and Environmental Sciences Faculty of Natural Sciences University of Stirling Stirling Scotland UK; ^2^ Laboratory of Proteomics and Microbiology Research Institute for Biosciences University of Mons Mons Belgium

**Keywords:** biofilm formation, marine biofilms, metaproteome, plastic debris, plastisphere

## Abstract

Marine plastispheres represent dynamic microhabitats where microorganisms colonise plastic debris and interact. Metaproteomics has provided novel insights into the metabolic processes within these communities; however, the early metabolic interactions driving the plastisphere formation remain unclear. This study utilised metaproteomic and metagenomic approaches to explore early plastisphere formation on low‐density polyethylene (LDPE) over 3 (D3) and 7 (D7) days, focusing on microbial diversity, activity and biofilm development. In total, 2948 proteins were analysed, revealing dominant proteomes from *Pseudomonas* and *Marinomonas*, with near‐complete metagenome‐assembled genomes (MAGs). *Pseudomonas* dominated at D3, whilst at D7, *Marinomonas*, along with *Acinetobacter, Vibrio* and other genera became more prevalent. *Pseudomonas* and *Marinomonas* showed high expression of reactive oxygen species (ROS) suppression proteins, associated with oxidative stress regulation, whilst granule formation, and alternative carbon utilisation enzymes, also indicated nutrient limitations. Interestingly, 13 alkanes and other xenobiotic degradation enzymes were expressed by five genera. The expression of toxins, several type VI secretion system (TVISS) proteins, and biofilm formation proteins by *Pseudomonas* indicated their competitive advantage against other taxa. Upregulated metabolic pathways relating to substrate transport also suggested enhanced nutrient cross‐feeding within the more diverse biofilm community. These insights enhance our understanding of plastisphere ecology and its potential for biotechnological applications.

**Abbreviations**
AD: alcohol dehydrogenase
Ahp: alkyl hydroperoxide reductase
ALDH: aldehyde dehydrogenase
ASW+G: artificial seawater + vitamins, trace metals and glucose
BHB+v: Bushnell Haas broth + vitamins and trace metals
CopA: copper resistance protein A
CSD: cold shock domain‐containing protein
D3/D7: Day 3/7
FC: fold change
FDR: false discovery rate
Fhp: Flavohemoprotein
HGT: horizontal gene transfer
Kat: catalase peroxidase
LDPE: low‐density polyethylene
MAG: metagenome‐assembled genome
Omp: outer membrane protein
ROS: reactive oxygen species
TelA: toxic anion resistance protein
TerD: tellurium resistance protein
TIISS/TVISS: type‐ II/VI secretion system
> D3/> D7: protein(s) upregulated on D3/D7

## 1 Introduction

Over 400.3 million metric tonnes of plastic are manufactured, utilised and discarded annually [1]. Despite diverse waste management strategies implemented by national governments and public initiatives aimed at controlling plastic waste, an estimated 19–23 million tonnes of plastic pollution enter the marine environment each year [2, 3]. Marine plastic debris, along with the resulting microplastics, is now anticipated to enter nearly all marine ecosystems, threatening the health of our oceans [4]. Direct studies of marine plastic debris reveal a complex relationship between plastics and the ocean's most abundant biogeochemical cyclers and producers: microorganisms [5, 6]. As natural biofilm‐forming organisms, and secondary surface colonisers, these microorganisms directly interact with marine plastic debris through the formation of the ‘plastisphere’ [7].

**Summary**

• Given the increasing plastic pollution in marine environments, understanding early plastisphere assembly is essential from both ecological and biotechnological perspectives. This study advances knowledge by identifying 13 pollutant‐degrading enzymes in addition to those found through prior metaproteomic research, shedding light on the bioremediative potential of multi‐species biofilms. Their expression by constitutive genera, particularly *Marinomonas*, also underscores the recognised association of these genera with environmental bioremediation. Additionally, interspecies competition and oxidative stress responses, shaped by resource limitations, were found to govern biofilm dynamics. The selection of generalist species and potential pathogens is concerning due to plastic's ability to travel through marine ecosystems. However, the cooperative behaviour among plastisphere members supports prior research demonstrating biofilms as resilient microbial solutions. This research not only deepens our understanding of microbial colonisation and interaction but also highlights the utility of metaproteomics in studying complex environmental communities. Insights from this study also contribute to the broader field of plastisphere ecology, offering pathways for future research into managing plastic pollution and developing biotechnological strategies for marine ecosystem resilience.

Marine plastispheres are complex, multi‐species biofilms that consist of both eukaryotic and prokaryotic producers, hydrocarbonoclastic organisms, pathogens and other biogeochemical cyclers [6, 7, 8, 9, 10]. The composition of these communities is influenced by several factors, including the type of plastic, its chemical additives, environmental location, seasonal changes and the time available for colonisation [6]. Among these factors, the duration of plastisphere development (i.e., the stage at which the plastisphere is collected) appears to exert the most consistent influence across studies. Microbial abundance and diversity are often low in the initial hours following colonisation, peak within 1–2 weeks and subsequently decline [10, 11, 12, 13]. Metaproteomic studies of early biofilm formation, though not specific to the plastisphere, have uncovered both competition and cooperation between species; interactions that are likely to be prevalent within the first days of plastisphere development [14, 15]. This early plastisphere is particularly significant, as it may be the only stage of biofilm development where direct microbe‐plastic interactions occur. As biofilms continue to form, new layers often cover older ones, shielding the upper biofilm layers from direct contact with the plastic surface [11].

Prior metaproteomic research on multi‐species biofilm formation [14, 15] and established marine plastispheres [8, 16, 17] has offered insights into how microorganisms interact with plastic substrates and with each other. A recent study by our research group also highlighted the importance of comparing new versus established plastispheres by studying their meta‐genome and proteome after 1‐ and 2‐weeks respective growth. This study demonstrated a significant shift in microbial diversity and composition whilst maintaining a core assemblage of active *Proteobacteria*, including *Marinomonas*, *Pseudomonas* and *Pseudoalteromonas* [9]. Additionally, this study highlighted the differential regulation of proteins involved in cellular attachment and energy metabolism during the first 2 weeks of colonisation, offering important context for understanding the evolution of biofilm dynamics. However, the molecular mechanisms driving the functioning of marine plastisphere communities in the earliest stages of development remain poorly characterised at the proteome level.

In the present study, our aim was to build on our previous research by providing a deeper understanding of the community structure of the young plastisphere and the metabolic activity of present microbial genera [9]. We employ metaproteomics to dissect the molecular basis of plastisphere formation within the first 7 days of colonisation, with a particular focus on the mechanisms associated with stress, biofilm‐formation, interspecies interaction and plastic biodegradation.

## 2 Materials and Methods

### 2.1 Recovery of Plastisphere Stock Communities

Beached plastics were collected from Oban Bay (56° 24’ 50.4’’ N, 5° 28’ 19.2’’ W; Scotland) on 6 August 2022 and washed in artificial seawater [18] to remove loosely attached organisms, prior to preservation at −20°C until use. For biofilm extraction, the transparent plastics within this collection were isolated from coloured plastics due to their close resemblance to the low‐additive, transparent low‐density polyethylene (LDPE) used in Section 2.2 [19]. To increase plastisphere biomass, the transparent plastics were placed into glass containers with 300 mL artificial seawater (24 g/L NaCl, 10.8 g/L MgCl_2_.H_2_O, 4 g/L Na_2_SO_4_, 1.5 g/L CaCl_2_.2H_2_O, 0.68 g/L KCl, 0.5 g/L NH_4_Cl, 0.2 g/L NaHCO_3_, 0.1 g/L KBr, 0.04 g/L NaHPO_4_, 0.025 g/L H_3_BO_3_, 0.024 g/L SrCl_2_.6H_2_O, 0.002 g/L NaF, PH 7.0) supplemented with vitamins, trace metals and glucose (ASW+G) and incubated with shaking (65 rpm) at 15°C for 3 days, equalling Oban Bay's highest seawater temperature. Excess ASW+G was carefully removed, and plastics were vortexed in the remaining media and rinsed with additional ASW+G to ensure the biofilm was fully detached. The plastics were then removed and the detached plastisphere cells were pelleted by centrifugation (14°C, 5000 *g*, 7 min), resuspended in 15 mL ASW+G and preserved with 30% glycerol at −80°C until further use.

### 2.2 Plastisphere Growth

**FIGURE 1:**
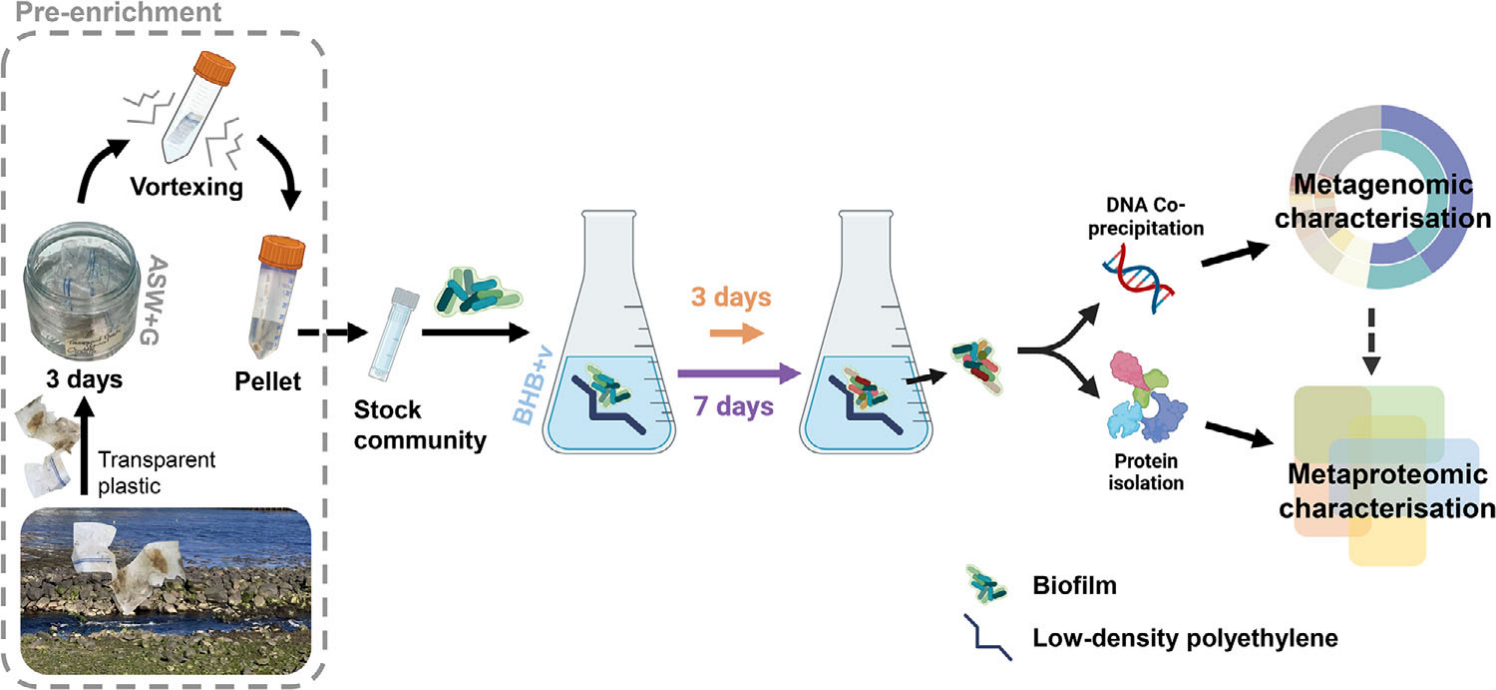
Three‐ and seven‐days growth of a marine microbial community on LDPE, and analysis of biofilm structure and functioning. ASW+G, artificial seawater supplemented with vitamins, trace metals, and glucose; BHB+v, Bushnell Haas Broth supplemented with vitamins and trace metals.

For this study, LDPE, one of the most widely produced plastics globally (45.7 Mt annually) [1], was selected as the substrate for plastisphere growth. Five pieces of LDPE (2 × 10 cm folded in/to 8ths; ET31‐FM‐000101; Goodfellow, England) per replicate were added to 150 mL of Bushnell Haas Broth supplemented with vitamins and trace metals (BHB+v) [20] and inoculated with 50 µL of plastisphere stock community. Each replicate (*n* = 4) was then incubated (65 rpm, 15°C) in 250 mL glass Erlenmeyer flasks for 3 or 7 days to monitor early plastisphere development through the later use of meta‐genomics and proteomics [11, 12, 13]. These incubation times were selected based on our previous findings [21], as they allow for biofilm formation, providing sufficient material for metaproteomic analysis, whilst also capturing the earliest stages of plastisphere formation [10] (Figure 1).

### 2.3 Protein Extraction

After incubation, protein was extracted from plastics and digested according to Messer et al. (16). Plastics were removed from media using forceps and allowed to dry under laminar flow. Once dry, plastics were bead beaten (1 mm glass beads) in a 2% sodium dodecyl sulphate (SDS) solution three times in 10‐min intervals. Cell media was then sonicated (1 s pulse, 1 s gap, 40% Amp.) twice, and centrifuged (8000 *g*, 10 min). The pellet was then sonicated and centrifuged again (as above). A quarter of the supernatant was preserved at −80°C for metagenomic analyses (Section 2.4), and the remaining supernatant (5–6 mL) was transferred to 3K centricons and centrifuged at 7000 *g* until ≤ 250 µL sample remained. Proteins were precipitated with cold acetone overnight at −80°C, with an acetone/aqueous protein solution ratio of 4:1. The protein pellet was resuspended in 6 M Guanidine HCl in dipotassium phosphate buffer, sonicated (1 s pulse, 1 s gap, 40% Amp.), then centrifuged (13,000 *g*, 15 min). The supernatant was then diluted with LC‐MS grade water, with a water/aqueous protein solution ratio of 1:1 and preserved at −80°C. Protein quantification revealed 35.13 mg (±0.67; *n* = 4), and 6.68 mg (±2.38; *n* = 4) of protein within the samples extracted after 3 (D3) and 7 (D7) days incubation respectively.

For protein digestion, 100 µg of protein per sample was reduced (10 mM 1,4‐Dithioerythritol), alkylated (25 mM iodoacetamide) and precipitated with acetone (acetone/aqueous protein solution ratio of 4:1) before being digested at 37°C overnight in 20 µL of sequencing grade modified trypsin (EC 232‐650‐8) as described previously [16].

Protein samples were analysed on an ultra‐high‐performance liquid chromatography–high‐resolution tandem mass spectrometer (UHPLC‐HRMS/MS) with an Eksigent NanoLC 400 and AB Sciex TripleTOF 6600 system. Two micrograms of peptides were analysed using acquisition parameters previously reported [22] in data‐dependent acquisition (DDA) mode. Mass spectrometry (MS/MS) runs were conducted with micro injection (75 min LC separation) modes.

### 2.4 Shotgun Metagenomics

DNA was co‐precipitated from the plastisphere samples for metagenomic analysis by adding protein precipitation solution (Promega, A7951) with a sample/protein precipitation solution ratio of 2:1, followed by centrifugation (20,379 *g*, 15 min). Filter‐sterilised 3 M sodium acetate (300 µL) was added to the supernatant, followed by > 95% cold ethanol at a sample/ethanol solution ratio of 1:2, and DNA was precipitated overnight (−20°C). The precipitated DNA was pelleted by centrifugation (20,379 *g*, 15 min), and washed twice using 70% ethanol, centrifuged again (16,000 *g*, 15 min), then briefly dried under laminar flow. DNA pellets were resuspended in 20 µL UltraPure water, heated to 55°C (5 min) to facilitate dissolution, vortexed and preserved at −80°C.

Metagenomic analysis was performed on the MinION (Mk1c; Oxford Nanopore Technologies; ONT), using the NEBNext Oxford Nanopore companion module and associated protocols to prepare the DNA sequencing library. Replicates were pooled to reach 800 ng, resulting in one sample per condition (D3 and D7) for input into the Native Barcoding Kit 24 (SQK‐NBD112.24). The resulting library was loaded onto the flowcell (FLO‐MIN106) according to the manufacturer instructions, and samples were sequenced for 24 h. Read lengths below 200 bp, and a quality score of 8 were discarded during subsequent real‐time basecalling in MinKnow (v22.10.5). This resulted in 6.72 Gb of estimated bases, and 5.66 million reads which were trimmed (minimum quality score 7) and normalised using BBDuk and BBNorm, respectively. These reads were taxonomically annotated using Kaiju [23], assembled (2955 total contigs) using MetaFlye [24], and functionally annotated with DRAM, leveraging KBase, as previously described [16].

### 2.5 Shotgun Metaproteomics

Protein searches were performed within ProteinPilot (v5.0.3.1029, 9521aa4603a; Paragon Algorithm: 5.0.3.1029, 1029; AB SCIEX) using the AB SCIEX OneOmics software package, and databases created from our combined metagenomic data (Section 2.4), and other publicly available databases for plastic metabolism (PlasticDB) [25], virulence factors (VFDB) [26] and antimicrobial resistance (CARD) [27]. To create a database of our combined metagenomic data, the genus‐level taxonomic (.txt) and functional (.faa) annotations (Section 2.4) were used in the mPies (v1.0) [28] database creation workflow (https://github.com/johanneswerner/mPies). Each database was then used to match spectral (DDA) data to taxonomic and proteomic data in ProteinPilot with a global false discovery rate (FDR) of 1%. The resulting files were then reprocessed with mPies using parameters described in Messer et al. (16) for protein grouping. This produced functionally annotated proteins, and corresponding taxonomic annotations after protein alignment with Diamond, within the mPies workflow [28]. All taxonomic data was subsequently filtered according to confidence score (≥ 80%), and proteins with < 2 associated peptides were removed. Before annotation, intensity‐based relative quantification between the conditions was also performed in Skyline (v. 22.2) using the following parameters: mass analyser TOF, normalisation method total ion current (TIC), centroided peak integration, MS1 tolerance 0.05 Da, MS2 tolerance 0.1 Da, maximum missed cleavages 2 and minimum peptides per protein 2. To identify significantly up‐ or down‐regulated proteins between D3 and D7, the MSstats package was leveraged within Skyline. An adjusted *p* value of < 0.05 and fold change (FC) of 1.5 was considered significant. Missing annotations caused by protein inference issues were manually curated using BLAST (https://www.uniprot.org/blast) if the same protein was matched repeatedly (> 50% matches), the highest‐rated peptide score equalled ≥ 100, there was a match at the species level or a combination of these criteria. Metagenomic data, database search exports, relative quantification data, peptide and protein data are provided in Supporting files S1: S6. Subsequent data wrangling and the production of figures was performed using RStudio (v. 4.2.2).

## 3 Results

Metaproteomic analysis of early plastisphere communities resulted in the identification of 2037 and 1886 proteins at D3 and D7 respectively, with corresponding spectral coverages of 33.85% and 28.1% (Supporting File S5). The majority of proteins in each dataset (D3 = 70.2%; D7 = 72.6%) were successfully annotated using the consensus approach employed by mPies, and the remaining 29.8% (D3) and 27.4% (D7) of proteins were annotated manually. Of the identified proteins, 1499 were non‐redundant at D3 and 1449 at D7 (2‐11 unique peptides per protein), of which only 1.85% (±0.05%) were taxonomically unknown or functionally uncharacterised after manual annotation. Overall, 97.4% (±1.2%) of proteins were taxonomically annotated to the phylum level, 96.5% (±0.8%) were annotated to class level, 93.4% (±0.5%) to the order level, 92.2% (±0.6%) to the family level, 87.5% (±3.7%) were annotated to the genus level, and 27.1% (±8.1%) of proteins were annotated to species level (Supporting File S1). However, a difference in the coverage of these annotations was observed between D3 and D7 metaproteomes, such that 90.9% of proteins were annotated to genus level at D3, compared to 84.1% at D7 and 35.2% of proteins were annotated to species level at D3, relative to 19% at D7. The relative quantification of identified proteins revealed that 711 non‐redundant proteins were shared across D3 and D7, with 387 of these found to be upregulated on D3 (representing 120 unique protein groups), and 323 were upregulated on D7 (comprised of 292 unique) (Supporting File S4). These results allowed detailed characterisation of the active taxa and their expressed functions during the early colonisation of plastic.

### 3.1 A Heterotrophic Marine Plastisphere

**FIGURE 2:**
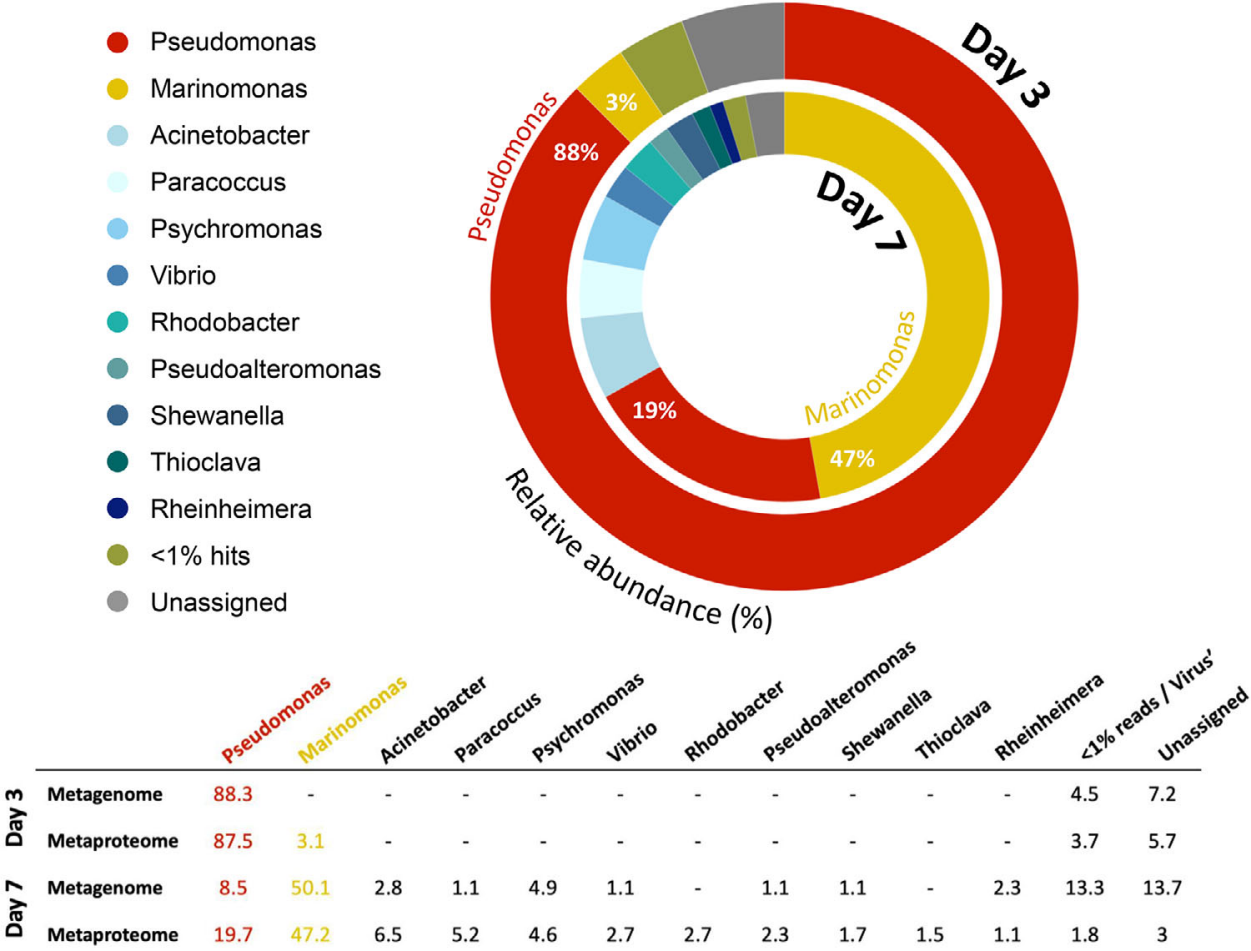
Relative abundance of microbial species found within the metaproteome at each time point. Calculated using the number of proteins associated with each genus relative to all the proteins found in that condition. Values used for the doughnut plot are shown in the table below, as well as corresponding metagenomic data.

At both time points, the majority of the bacterial community belonged to the class Gammaproteobacteria (> 85%), with an increased relative abundance of Alphaproteobacteria (1.1%–10.3%) and Bacteroidetes (0.05%–0.35%) between D3 and D7 (Figure 2). Within Gammaproteobacteria, two high‐quality metagenome assembled genomes (MAGs) were recovered for the genera *Pseudomonas* and *Marinomonas*, with a completeness of 98.08% and 96.54%, respectively. Within these MAGs, 1668 and 979 proteins (1% FDR) were identified for *Pseudomonas* and *Marinomonas* respectively (Supporting File S3).

Upon examination of our combined metagenomic and metaproteomic data at the genera level, both the composition and activity of the community exhibited a marked shift between D3 and D7. Initially dominated by active *Pseudomonas* at D3, the plastisphere transitioned into a more diverse community by D7, where *Marinomonas*, *Pseudomonas*, *Acinetobacter*, *Paracoccus*, *Psychromonas*, *Vibrio*, *Rhodobacter*, *Pseudoalteromonas* and other minor genera were present in greater abundance (Figure 2). All active bacterial genera identified in this study were mainly facultative heterotrophs. Four bacterial autotrophs, photoheterotrophs and mixotrophs (*Rhodobacter*, *Photobacterium*, *Sinirhodobacter* and *Sinorhizobium*) were active on both D3 and D7, though no proteins detected for these minority genera were associated with autotrophic processes.

This heterotrophic nature of the biofilm was evidenced by the expression of key metabolic enzymes, including succinate dehydrogenase, citrate synthase, malate dehydrogenase and isocitrate dehydrogenase — all integral to the tricarboxylic acid (TCA) cycle — as well as glyceraldehyde‐3‐phosphate dehydrogenase and enolase, which are involved in carbohydrate metabolism and ATP synthase, which is involved in energy production (Supporting File S4). These metabolic proteins were among the most ubiquitously expressed across both time points, encompassing 10 of the 20 active organisms in this study.

### 3.2 Key Functional Categories Driving Early Plastisphere Development

**FIGURE 3:**
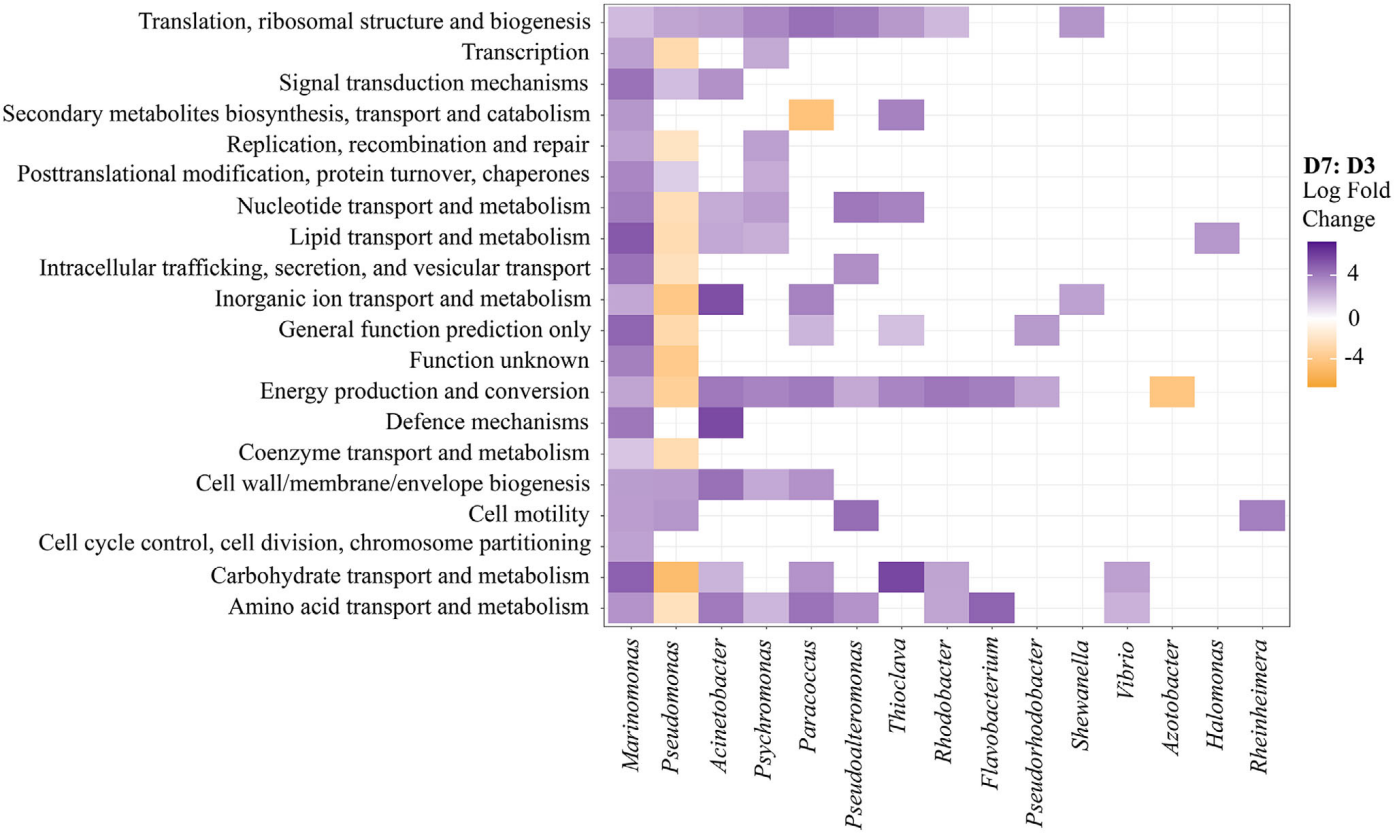
Total differentially regulated proteins (FC > 1.5, *P*‐adj. < 0.05) according to function and taxonomic classification. Positive FC (purple) indicates greater abundance of proteins on D7 (purple), and negative FC (orange) indicates greater expression on D3. FC indicates fold changes

The proteins expressed in D3 and D7 were classified into functional categories, with ‘translation, ribosomal structure and biogenesis’, ‘energy production and conversion’ and ‘amino acid transport and metabolism’ being the most abundant across most genera (Figure 3a). These categories were then followed by functions associated with ‘cell wall/membrane/envelope biogenesis’, ‘cell motility’ and biofilm formation.

Within ‘energy production and conversion’, proteins linked to the TCA cycle were prominent and differentially expressed between time points. These include succinate dehydrogenase, expressed by *Pseudomonas* (> D3; FC 2.83), *Marinomonas* (> D7; FC 4.53), *Paracoccus* (> D7; FC 2.72), *Acinetobacter* (> D7; FC 4.27) and *Vibrio* on both days, as well as by *Rhodobacter* and *Shewanella* on D7. Additionally, citrate synthase was expressed by *Pseudomonas* (D3; D7), *Marinomonas* (D7), *Acinetobacter* (D7) and *Psychromonas* (D7), whilst malate dehydrogenase was expressed by *Pseudomonas*, *Marinomonas* (> D7; FC 3.6), *Paracoccus* (> D7; FC 2.92) and *Pseudoalteromonas* (> D7; FC 4.13) on both days. Isocitrate dehydrogenase was expressed by *Pseudomonas* (> D7; FC 3.01 ± 0.79), *Marinomonas* (> D7; FC 3.21 ±0.52), *Psychromonas* (> D7; FC 3.85) and *Flavobacterium* (> D7; FC 4.07) (Supporting File S4).

Interestingly, proteins associated with ‘amino acid transport and metabolism’ were differentially regulated across nine genera (Figure 3a), accounting for 11.5% (± 1.5) of the total differentially regulated proteins. These include enzymes involved in glutamate cycling (e.g., glutamate methylesterase, > D7 *Marinomonas*, FC 3.03; glutamine synthetase, > D7 *Marinomonas*, FC 2.7; > D7 *Paracoccus*, FC 2.1), arginine cycling (e.g., acetylornithine aminotransferase, > D7 *Marinomonas*, FC 3.79) and the regulation and transport of branched‐chain amino acids (e.g., acetohydroxy‐acid isomeroreductase, > D7 *Flavobacterium*, FC 4.95).

**FIGURE 4:**
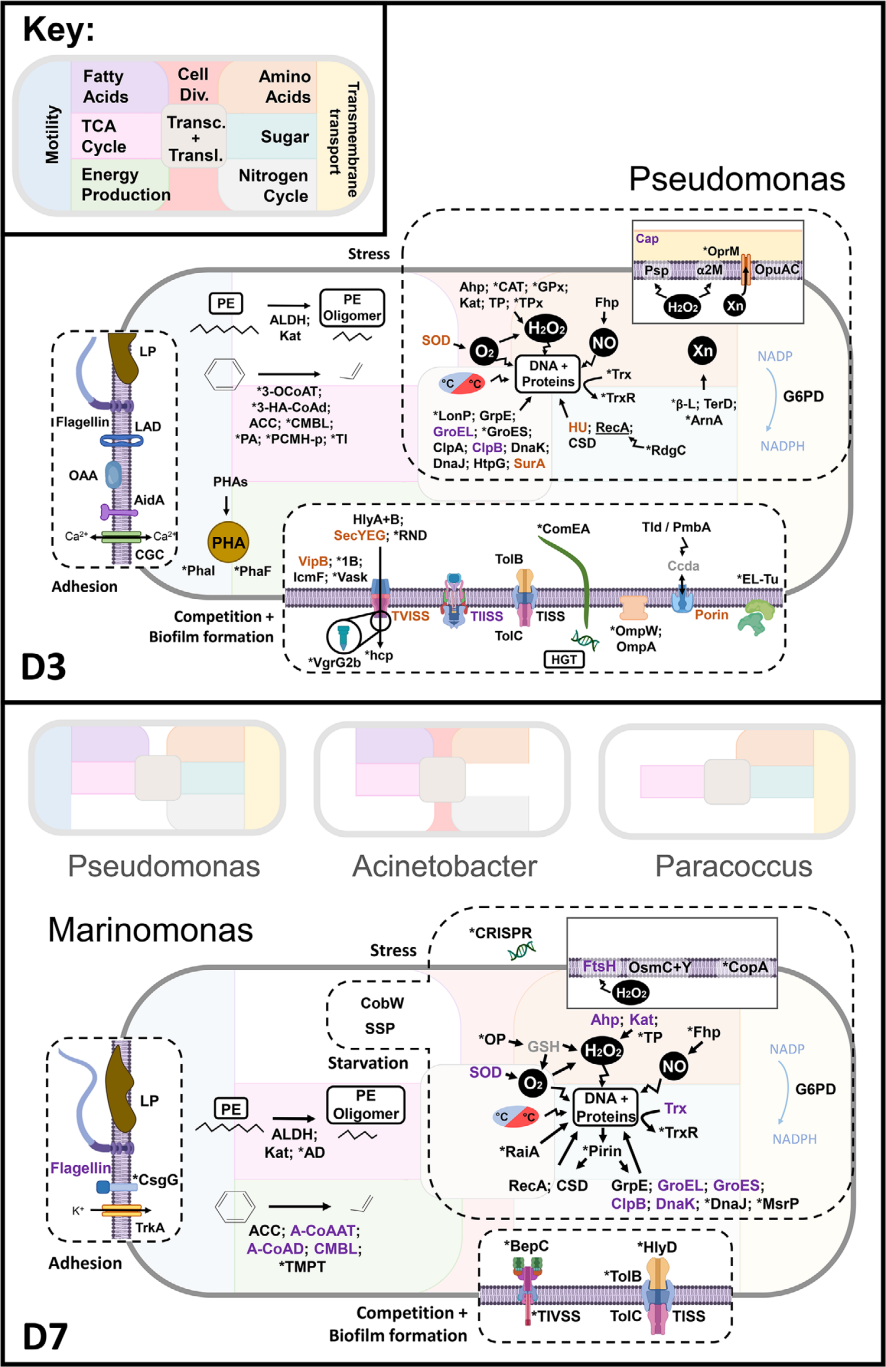
Graphic representation of biologically relevant processes found within this study's most abundant bacterial genera from relative quantification and qualitative analysis of the metaproteomic dataset. Purple = more expressed on Day 7, orange = more expressed on Day 3, black = qualitative results, * = qualitative results with 1 entry, grey = not found, but can be assumed. Colour code indicates all basal processes active within the genus at that time (> 1 protein each). All basal processes active in the largest, annotated microorganisms. **Figure elements**: H2O2 indicates hydrogen peroxide; HGT, horizontal gene transfer; NO, nitric oxide; O2, superoxide; PE, polyethylene; PHA, polyhydroxyalkanoate granule; Xn, xenobiotic. **Stress** (osmotic, oxidative, heat/cold, interspecies interaction, xenobiotic): Ahp, alkyl hydroperoxide reductase; ArnA, polymyxin resistance protein; α2 M, alpha‐2‐macroglobulin; β‐L, β‐lacatamse; Cap, capsular biosynthesis protein; CAT, catalase; CopA, copper resistance protein A; CSD, cold shock domain containing protein; Fhp, flavohemoprotein; FtsH, ATP‐dependent zinc metalloprotease; G6PD, glucose‐6‐phosphate dehydrogenase; Gpx, glutathione peroxidase; GrpE, DnaK nucleotide exchange factor; GSH, glutathione; HU, transcriptional regulator HU; Kat, catalase peroxidase (also PE degradation); LonP, lon protease; MsrP, methionine sulfoxide reductase; OP, 5‐oxoprolinase; OprM, outer membrane efflux system protein; Osm, osmotically inducible protein; Psp, phage shock protein; RdgC, recombination‐associated protein; RecA, SOS response protein; SOD, superoxide dismutase; OpuAC, substrate binding domain of ABC‐type glycine betaine transport system; TerD, tellurium resistance protein; TP, thiol peroxidase; TPx, thioredoxin peroxidase; Trx, thioredoxin; TrxR, thioredoxin reductase; Chaperones‐ ClpA, ClpB, DnaJ, DnaK, HtpG, SurA; Chaperonins‐ GroEL, GroES. **Virulence and biofilm formation**: 1B, hemolysin co‐regulated protein; BepC, outer membrane efflux protein; Ccda, control of cell death protein; ComEA, competence protein; EL‐Tu, elongation factor Tu; hcp, hemolysin‐coregulated protein; Hly, hemolysin activation/ secretion protein; Omp, outer membrane protein; RND, efflux resistance‐nodulation‐division (RND) transporter; SecYEG, translocon; TISS, type‐I secretion system; TIISS, type‐II secretion system; TIVSS, type‐IV secretion system; TVISS, type‐VI secretion system; VgrG2b, actin cross‐linking spike protein; Translocons‐ IcmF, Tld/PmbA, Vask, VipB. **Starvation**: CobW, cobalamin biosynthesis protein; PhaF, polyhydroxyalkanoate (Pha) granule‐associated protein F; Phal, Pha granule‐associated protein L; PHAs, PHA synthase; SSP, stringent starvation protein. **Adhesion**: AidA, adhesin; CGC, calcium‐gated channel; CsgG, curli assembly protein G; LAD, large adhesive protein; LP, lipoprotein; OAA, OAA‐family lectin sugar binding domain containing protein; TrkA, Potassium uptake protein. **Aromatic hydrocarbon degradation**: 3‐OCoAT, 3‐oxoadipate‐CoA transferase; 3‐HA‐CoAd, 3‐hydroxyacyl‐CoA dehydrogenase; ACC, acetyl‐CoA carboxylase; A‐CoAAT, acetyl‐CoA acetyltransferase; A‐CoAD, acetyl‐CoA dehydrogenase; CMBL, dienelactone hydrolase; PA, pyruvate adolase; PCMH‐p, p‐crestol methylhydroxylase (PCMH) type protein; TI, thiolase I; TMPT, thiopurine s‐methyltransferase. **PE degradation**: AD, alcohol dehydrogenase; ALDH, aldehyde dehydrogenase.

Within the categories of ‘cell wall/membrane/envelope biogenesis’ and ‘cell motility’, several membrane proteins facilitating substrate binding and biofilm formation were expressed. Key adhesive structures include lipoprotein (expressed by *Pseudomonas* on D3 and *Marinomonas* on D7), large adhesive protein (LAD; expressed by *Pseudomonas* on D3) and outer membrane protein A (OmpA; expressed by *Thioclava*, *Shewanella*, *Pseudomonas*, *Rheinheimera* on D7), with *Acinetobacter* showing the highest expression on D7 (FC 4.4 ± 0.14; Figure 3). Additionally, Type V secretory adhesin AidA (FC 2.92 ± 0.76) and elements of the Type II secretion system (TIISS) were expressed by *Pseudomonas* on D3 (Figure 4). Pili were also expressed by *Pseudomonas* on both D3 and D7, with pili assembly proteins PilZ (*Pseudomonas*, D3) and FimV (*Marinomonas*, D7) similarly expressed. In parallel, the curli assembly/transport component CsgG was identified in *Marinomonas* on D7 (Figure 4), indicating potential involvement in biofilm formation (Figure 4). Additionally, flagellin, which may facilitate substrate adhesion, exhibited higher expression on D7 compared to D3. This increase was particularly noted in *Marinomonas* (FC 4.86 ± 0.79), *Pseudoalteromonas* (FC 4.5 ± 0.13), *Rheinheimera* (FC 4.02) and *Pseudomonas* (FC 2.96 ± 0.32). However, certain *Pseudomonas* species demonstrated elevated flagellin expression on D3 (FC 3.35 ± 0.31), suggesting variation in flagellar activity between strains across different time points.

Proteins associated with biofilm arrangement including components of calcium‐gated (EF‐hand domain‐containing protein, dCache_2 domain‐containing protein, *Pseudomonas*), and potassium channels (potassium uptake protein TrkA, *Marinomonas*) were expressed on D3 and D7, respectively. One instance of quorum sensing, via the expression of the autoinducer 2‐binding periplasmic protein LuxP, was found on D7, expressed by an uncharacterised species of *Vibrio*. Further examples of cell‐cell interactions were identified through the expression of competence protein ComEA (*Pseudomonas*, D3), involved in horizontal gene transfer (HGT), the OAA‐family lectin‐sugar binding domain‐containing protein (> D3 *Pseudomonas*, FC 4.72) and TIVSS protein (*Marinomonas*, D7). Proteins involved in purine nucleotide biosynthesis (e.g., bifunctional purine biosynthesis protein PurH), which are also thought to play a role in early biofilm formation, were expressed on both days (Figure 3; Supporting file S4).

### 3.3 Stress‐Driven Resilience and Competition in a Nascent Marine Plastisphere

Proteins associated with the regulation of oxidative stress, nutrient limitation and interspecies competition were abundantly expressed, reflecting this community's resilience to environmental stress and intense competitive dynamics (Figure 4). Evidence of nutrient starvation, particularly of carbon, was observed through the expression of cobalamin biosynthesis protein, CobW (*Marinomonas*) and stringent starvation protein (*Marinomonas, Psychromonas*) on D7. Additionally, poly(3‐hydroxyalkanoate) granule‐associated proteins PhaF, Phal and poly(R)‐hydroxyalkanoic acid (Pha) synthase expressed by *Pseudomonas* on D3 (Figure 4), provided evidence for starvation responses due to the limitation of other nutrients (viz. nitrogen, phosphorus). Moreover, evidence for alternative carbon source utilisation was observed, including three enzymes associated with the alkane degradation pathway. Including, catalase‐peroxidase (Kat) expressed by *Marinomonas* (> D7; FC 2.96), *Psychromonas* (D7) and *Pseudomonas* (D3), aldehyde dehydrogenase (ALDH) expressed by *Pseudomonas* (D3; D7), *Thioclava* (D7), *Rhodobacter* (D7) and *Marinomonas* (D7), and alcohol dehydrogenase (AD) expressed by *Marinomonas* (D7; Figure 4). Protein identification using PlasticDB—a database containing proteins specifically mediating plastic biodegradation—identified the polyethylene‐degrading enzyme laccase expressed by *Psychromonas*, on D7 (Figure 2; Supporting file S2).

Proteins associated with carbon metabolism, under the functional classification ‘carbohydrate transport and metabolism’ (Figure 3), were consistently upregulated by *Pseudomonas* on D3, and by *Marinomonas* on D7. CreA family proteins and response regulatory domain‐containing proteins were also expressed by *Pseudomonas* on D3. Tricarboxylic transporters were expressed by *Pseudomonas* on D3, and by *Pseudomonas, Pseudorhodobacter, Rhodobacter* and *Marinomonas* on D7 (Supporting file S4). Interestingly, enzymes involved in aromatic hydrocarbon biodegradation were characterised, including dienelactone hydrolase, mostly expressed by *Marinomonas* on D7 (FC 2.03; Figure 3), and by *Pseudomonas* on D3. Nine other enzymes associated with this process were discovered on both days, including 3‐oxoadipate‐CoA transferase, 3‐hydroxyacyl‐CoA dehydrogenase, acetyl‐CoA carboxylase, acetyl‐CoA acetyltransferase, acetyl‐CoA dehydrogenase, pyruvate aldolase, p‐cresol methylhydroxylase (PCMH)‐type protein, thiolase I and thiopurine s‐methyltransferase. Of these, acetyl‐CoA acetyltransferase and acetyl‐CoA dehydrogenase were most expressed on D7 (FC 4.51 and 2.48, respectively). Evidence for nitrogen cycling was found through the constitutive expression of nitrate reductase (*Pseudomonas*, D3; *Pseudomonas, Marinomonas*, D7), nitrous‐oxide reductase (*Pseudomonas*, D7) and nitrogen regulatory protein PII (*Pseudomonas*, D3; *Pseudomonas, Acinetobacter*, D7) on D3 and D7. Nitrogen and urea transporters were also expressed by *Pseudomonas, Marinomonas* and *Paracoccus* on both days.

In response to intracellular ROS, the plastisphere communities expressed a variety of stress‐response proteins, including alkyl hydroperoxide reductase (Ahp), catalase (CAT), catalase peroxidase (Kat), glutathione peroxidase (Gpx), thiol peroxidase (TP) and thioredoxin peroxidase (TPx) in response to hydrogen peroxide (H_2_O_2_), flavohemoprotein (Fhp) to respond to nitric oxide (NO), and superoxide dismutase (SOD) to neutralise superoxide radicals. The most frequently expressed stress‐response proteins were Ahp (*Pseudomonas*, D3; *Pseudomonas*, *Paracoccus*, D7; > D7 *Acinetobacter*, FC 5.83; > D7 *Marinomonas*, FC 4.32), Fhp (*Pseudomonas*, D3; *Acinetobacter*, D7; > D7 *Marinomonas*, FC 3.5), Kat (*Pseudomonas*, D3; *Psychromonas*, D7; > D7 *Marinomonas*, FC 2.96) and SOD (*Paracoccus*, D3; *Acinetobacter*, *Psychromonas*, *Rhodobacter*, D7; > D3 *Pseudomonas*, FC 3.92; > D7 *Marinomonas*, FC 2.74) due to their consistent expression across multiple genera (Figure 4; Supporting file S4). In response to metals and anions, *Marinomonas* (D7) and *Paracoccus* (D7) expressed copper resistance protein A (CopA), and toxic anion resistance protein (TelA), respectively, and *Pseudomonas* (D3) expressed tellurium resistance protein (TerD) (Figure 4). Osmotic stress proteins OsmC (*Pseudomonas, Marinomonas*, D7), and glucans biosynthesis protein C (*Pseudomonas*, D3) were also found. The downstream effects of stress were apparent in the expression of membrane repair proteins ATP‐dependent zinc metalloprotease (FtsH), α‐2 macroglobulin (α2 m) and phage shock protein (Psp). Additionally, transcriptional response proteins such as HU, RecA, RdgC, cold shock‐domain (CSD) proteins and DNA repair proteins such as GrpE, Lon protease and methionine sulfoxide reductase were also abundant. Further stress mitigation was supported by thioredoxin/thioredoxin reductase systems, along with a suite of chaperones and chaperonins (e.g., ClpA, ClpB, DnaJ, DnaK, GroEL, GroES, HtpG, SurA), and the redox enzyme glucose‐6‐phosphate dehydrogenase (Figure 4). Among these, the cold shock‐domain protein was the most abundantly expressed protein, found in the proteomes of *Pseudomonas* (> D3 FC 3.8), *Marinomonas* (D7), *Acinetobacter* (D7), *Paracoccus* (D7) and *Shewanella* (D7). Further protective mechanisms were also identified, such as dipicolinate synthase expression by *Pseudomonas* on D3 and capsular biosynthesis protein on D7. Interestingly, proteins linked to reactive oxygen species (ROS) generation, including sarcosine oxidase (*Pseudomonas*, D3) and Na(+)‐translocating NADH‐quinone reductase (*Marinomonas*; > D7 Marinomonas, FC 7.01), were also expressed.

Competition for resources was evidenced by the expression of proteins associated with competitive stress. This included the type VI secretion system (TVISS) proteins, such as TssM, contractile sheath large subunit, and secretion proteins Evp, IcmF and VasK, expressed by *Pseudomonas* (D3; FC 1.74), *Pseudoalteromonas* (D7; FC 3.54), and Acinetobacter (D7) (Figure 4; Supporting file S4). Competitive advantage was also indicated by the expression of the actin cross‐linking toxin VgrG2 by *Pseudomonas* on D3. Hemolysin‐related proteins, which may have been similarly involved in this competitive response, were also expressed by *Pseudomonas* at this time‐point (Figure 4). In potential response to antibiotic production, resistance mechanisms were expressed, such as polymyxin resistance protein and outer membrane efflux system protein by *Pseudomonas* on D3, and *β*‐lactamase by *Pseudomonas* and colicin‐I receptor proteins by *Shewanella* on D7.

Interestingly, the potential pathogens *Pseudomonas syringae* and *Pseudomonas aeruginosa*, expressed two TVISS proteins on D3, whilst *P. syringae* also expressed tol‐pal system proteins and a FeADH domain‐containing protein. These results indicate a complex competition network within the biofilm, where both resource limitation and interspecies antagonism shape the microbial community structure and resilience.

## 4 Discussion

In this study, we analysed 2948 non‐redundant proteins from the LDPE plastisphere, providing near‐complete proteomes for the two dominant genera *Pseudomonas* and *Marinomonas* within the first days of colonisation. Importantly, whilst metagenomic analysis reveals the genetic potential of microbial communities, the use of metaproteomics provided distinct advantages by enabling direct observation of enzymes expressed by bacteria at the plastic‐biofilm interface. This approach allowed us not only to confirm gene expression but also to precisely link specific proteins to their active roles, bridging the gap between genetic potential and functional protein production [29]. This extensive dataset offered valuable insights into the metabolic activity of these key taxa, as well as the broader functioning of the young plastisphere community. Through this approach, we revealed the intense competition between taxa within the rapidly developing community and identified the molecular mechanisms which may support plastisphere resilience to nutrient limitation and oxidative stress. Our results also revealed molecular interactions between the early plastic colonisers and the plastic itself, with important implications for the modulation of biofilm formation and the exploration of plastic biodegradation. These results provide new insights into the dynamics of the nascent plastisphere and their biotechnological potential.

### 4.1 Dominance of Key Taxa in the Young Marine Plastisphere

The activity of the young marine plastisphere was dominated by heterotrophic bacteria, particularly Gammaproteobacteria, consistent with our previous studies examining plastisphere formation and function [6, 9, 16], and with studies investigating the taxonomic diversity of the plastisphere [7, 30, 31]. Herein and in our previous work, plastisphere samples were collected from the Scottish coast, and biogeographic location appears to play a significant role in shaping plastisphere communities, as noted in several studies including our own [6, 9, 16]. Community development in the present study mirrored previous observations demonstrating that plastisphere diversity increases over time [10, 11, 12, 13]. Indeed, we observed a plastisphere community whose activity was dominated by *Pseudomonas* after 3 days of colonisation, shifting to an active community comprised of > 8 active genera, including Alphaproteobacteria and Bacteroidetes after 7 days. *Pseudomonas*, the pioneering genus in this study and one of two high‐quality MAGs recovered from the marine plastisphere, is known for its metabolic versatility and biotechnological potential. Its species span biogeochemical cyclers, symbionts, decomposers, denitrifying bacteria and pathogens [32]. This genus is particularly known for its biofilm‐forming capabilities, facilitated by the secretion of specialised membrane proteins and secretion systems which likely facilitate interactions with plastic (Section 4.3) [33]. We have previously observed *Pseudomonas* within the marine plastisphere, but at < 25% of the active community [9, 16], indicating that the functional role of *Pseudomonas* may be most significant within the initial stages of plastisphere development. As such, the expressed proteome of this organism provides important insights into microbe‐plastic interactions at the time of most significance, where colonisers specifically attracted to the plastic surface may utilise it as a substrate [34]. Nevertheless, the most abundant and active genera found on D7, *Marinomonas*, was the second high‐quality MAG recovered and is also a marine‐adapted genus typically associated with pollutant bioremediation [35, 36]. Its enrichment in this study suggests that the LDPE, and this study's restrictive growth conditions facilitated its growth and biofilm formation as a secondary coloniser. Consistent with this, in our previous work, *Marinomonas* was also the dominant and active taxa following 1–2 weeks LDPE colonisation, but it was less important within the mature marine plastisphere [9]. Additionally, further bioremediative genera such as *Thioclava* [30] were active on D7 (relative abundance 1.5%), alongside the presence of potential pathogens *P. aeruginosa*, and *P. syringae* which represented 4.6% and 17.5% of all annotated species, equalling 285 non‐redundant proteins [37, 38]. Indeed, *P. syringae* was the most annotated species in the D7 plastisphere, indicating a concerning dominance of potential pathogens within the portion of the plastisphere (19%) that could be annotated to this level. The observed diversity in this early‐stage plastisphere highlights the biofilm's dynamic nature, whilst the enrichment of two taxa as high‐quality MAGs underscores the selectivity of the plastisphere and this study's conditions, likely shaped by competitive interactions.

### 4.2 Biofilm Formation

Biofilm formation is a key survival strategy for microbial proliferation in challenging, oligotrophic marine environments [39, 40]. Several proteins involved in surface adhesion were found to be repetitively identified across different taxa known for their propensity to form biofilms. In this way, biofilm formation might have been promoted in *Pseudomonas*, *Marinomonas*, *Acinetobacter* and *Vibrio* through the expression of secretion systems I, II and IV, as well as flagellin, adhesin, curli, Omps, lipoprotein and large adhesive protein [41, 42, 43]. These surface adhesion mechanisms were predominantly expressed by *Pseudomonas*, potentially aiding their dominance and rapid colonisation of plastic at D3. The succession of *Marinomonas* after the initial colonisers further supports their biofilm‐forming ability, which is well‐documented in marine environments [36]. The detection of quorum‐sensing molecules expressed by *Vibrio* on D7 also indicates that this genus was involved in processes potentially related to surface adhesion, such as the regulation of membrane‐sorting proteins, which are often associated with coordinated biofilm development [44].

Biofilm formation may have also been supported by the upregulation of purine biosynthesis, the TCA cycle, arginine and glutamate cycling proteins, which are crucial for cross‐feeding within the biofilm community [39, 40, 45]. Moreover, our previous findings highlighted the critical role of glutamine in biofilm proliferation, likely due to this cross‐feeding [15, 16, 46]. Its regulation was found to be closely linked with ammonium availability and oxidative stress within carbon‐deficient environments, with *Marinomonas* showing distinct glutamine metabolism within the plastisphere [9]. This suggested a differential nutrient‐utilisation strategy between biofilm‐associated and planktonic communities, further supporting the notion of a resilient and metabolically adaptive plastisphere biofilm [16]. The observed increase in microbial diversity over time implies that the resources produced by pioneering species (e.g., carbon, amino acids) may have facilitated the growth of subsequent colonisers, creating a more complex and resilient biofilm [15, 46].

Importantly, plastisphere biofilms offer distinct advantages to microorganisms in these environments. For instance, the expression of TIVSS and a competence protein suggests the potential for HGT within the plastisphere [41], allowing microbes to share adaptive traits. Moreover, the expression of calcium‐gated channels (e.g., EF‐hand and dCache_2 domain‐containing proteins) and potassium uptake proteins (e.g., TrkA) further indicates that nutrients may have been exchanged between neighbouring cells, further enhancing cross‐feeding, and the biofilm's capacity to thrive under nutrient‐limited conditions [47]. Moreover, nitrogen sufficiency is suggested by the expression of nitrogen cycling proteins like nitrate reductase, nitrogen regulatory protein PII and glutamine. On the other hand, carbohydrates and amino acids were metabolised and transported within the community, further suggesting possible resource sharing through symbiosis or cross‐feeding, contributing to the overall resilience and stability of the biofilm [15, 46].

However, it remains unclear to what extent biofilm formation on plastic confers unique advantages over microbial communities in the surrounding seawater. Our previous research has suggested that the proteins associated with biofilm‐formation and cross‐feeding are enriched in plastisphere communities [9]. Though in this study of the nascent plastisphere, it is possible that the observed shifts in community composition (Section 4.1) may also reflect similar dynamics in seawater communities, where these same proteins could also be induced. Consequently, whilst the proteins expressed here are associated with biofilm formation and nutrient cross‐feeding in other studies, their specific roles in supporting biofilm structure and interspecies interactions on plastic are not fully resolved in this nascent community. Moreover, it is important to note that biofilm communities are not only cooperative but also competitive, as microorganisms compete for dominance and resources within these complex ecosystems.

### 4.3 Resilience and Competition

Proteins associated with the regulation of metabolic stressors were among the most abundant expressed non‐housekeeping proteins found in our datasets. Enzymes responsible for mitigating oxidative stress were frequently expressed by multiple genera (e.g., *Pseudomonas*, *Marinomonas*, *Acinetobacter*, *Paracoccus*, *Psychromonas* and *Rhodobacter*), with nearly the entire process of reactive ROS suppression (antioxidant production → DNA regulation → DNA repair) being characterised [48]. Oxidative stress in multi‐species biofilms may be triggered by spatial constraints, forced oxygen gradients, competition, nutrient limitation and the accumulation of abiotic stressors such as metals (e.g., CopA, TelA, TerD) [6, 49]. The plastic itself could also contribute to oxidative stress through the accumulation of ROS under UV‐light exposure [50, 51]. The multifunctionality of proteins identified here, such as the frequently expressed CSD protein (*Pseudomonas*, *Marinomonas*, *Acinetobacter*, *Paracoccus*, *Shewanella*), suggests that they may have been induced by a combination of these stressors. Indeed, in addition to their role in cold adaptation, cold shock proteins are crucial for maintaining protein stability and proper folding under stress conditions, extending their protective function against oxidative and metabolic stress [52].

Granule storage, competition and the expression of carbon‐limitation‐related proteins highlight nutrient stress during both sampling days. Interestingly, granule formation is often triggered by the scarcity of nitrogen and phosphorous, and an excess of carbon [53]. This nutrient limitation, particularly of carbon and energy sources, is known to cause dysregulation of core metabolic processes, leading to the accumulation of ROS, as well as an increased competition for resources [54, 55]. In support of this, the expression of VgrG2, which has roles in virulence, and inducing morphological defects in competing bacteria, may suggest competitive interactions within the plastisphere biofilm [56]. The production of this toxin, alongside other virulence factors (i.e., hemolysin), may have therefore induced membrane damage in plastisphere microorganisms adjacent to *Pseudomonas* through the action of the TVISS [56, 57]. Interestingly, we identified a near‐complete annotation of the TVISS machinery (i.e., TVISS proteins IcmF, VasK, VipB, membrane subunit TssM, contractile sheath small subunit, and large subunit, Hcp1 family TVISS effector, EvpB family TVISS protein) expressed by active members of the marine plastisphere [8, 16, 17]. These findings suggest that interspecies competition within the biofilm could be a significant factor in shaping planktonic community structure and dynamics. The expression of these virulence factors (i.e., hemolysin‐related proteins) within the nascent marine plastisphere also emphasises the proposed hazards of plastic debris as a carrier of potential pathogens [57, 58].

Using the TVISS as an indication of virulence [59], *Pseudomonas* were most competitive on D3 within a predominantly single genera biofilm. Previously, *Pseudomonas* and *Pseudoalteromonas* have also been found to outcompete other microbial species through the secretion of toxins [60, 61, 62], perhaps actively antagonising other members of the present microbial community. The expression of proteins used to convey antibiotic resistance (i.e., b‐lactamase, efflux RND transporters, capsule protein) [41, 62, 63] by *Pseudomonas* and *Marinomonas* on D7, highlights the intense competition within diverse microbial communities, which may shape the plastisphere. These mechanisms are important for maintaining cell integrity in a stressful environment, but their expression puts great demand on the cell's intracellular resources [41, 54, 59, 60], including those which may be used in key metabolic processes. The deregulation of these processes can further precipitate oxidative stress—similar to nutrient deprivation [54, 55]—and limit the growth of impacted bacteria [48]. Strategies used for interspecies competition by plastisphere, and also by planktonic microorganisms may have therefore contributed to this plastisphere's function and composition, though the influence of the surrounding planktonic community was not investigated here.

### 4.4 Metabolic Versatility for Plastic Degradation

In addition to the proteins which may have increased carbon utilisation efficiency in these plastispheres (Section 4.3), we identified proteins linked to the utilisation of alternative carbon sources, which is particularly relevant for plastic biodegradation. We do not find this to be conclusive evidence of pollutant biodegradation within these plastispheres. However, it is interesting to note that the two enzymes expressed here which align with Delacuvellerie's depiction of the alkene degradation pathway (AD, ALDH) are expected to be involved in the initial depolymerisation process of polyethylene degradation by Tao [8, 64]. Kat, also found here, but not in the alkane degradation pathway, is expected to play a role in the first steps of polyethylene biodegradation. Notably, this enzyme was expressed by *Pseudomonas*, a genus frequently associated with polyethylene biodegradation [65, 66, 67]. Our recent paper on the late‐stage plastisphere also revealed the expression of acyl‐CoA dehydrogenase [16], another element of the alkene degradation pathway, which was also expressed in Delacuvellerie's study alongside ferredoxin and ferredoxin reductase [8]. In combination, five enzymes associated with the alkene degradation pathway have now been annotated from within the marine plastisphere, plus an additional enzyme which may directly depolymerise polyethylene.

In alignment with our previous investigation, 10 proteins involved in fatty acid beta‐oxidation (i.e., acetyl‐CoA carboxylase, acetyltransferase and dehydrogenase) were found in these plastispheres [16]. This process, like the alkene degradation pathway, has been linked to both plastic biodegradation and the biodegradation of other xenobiotic hydrocarbons due to the need to dismantle their similarly structured hydrocarbon base [68]. Metabolites from xenobiotic degradation are also likely funnelled directly into the fatty acid beta‐oxidation pathway once initially depolymerised [64]. The dienelactone hydrolase enzyme expressed here is not linked to this pathway but is associated with the final stages of aromatic hydrocarbon (chlorophenol) degradation [69, 70]. The discovery of such enzymes is interesting because no such xenobiotics were present in these samples, and the plastics used were purportedly free of additives. Another trend of note is the upregulation of these proteins by *Marinomonas*. Even if these organisms did not actively biodegrade aromatic hydrocarbons in these samples, this may reveal a little of their adaptability to other xenobiotics such as crude oil in the marine environment [35].

The combined expression of enzymes which may be used by bacteria to biodegrade plastics, and other noted xenobiotics here may not indicate that plastic biodegradation is occurring in this scenario. However, it may suggest that organisms found here and in other marine plastispheres have the capacity to perform this biodegradation. Interestingly, the incomplete annotation of these processes can rarely be attributed to a singular genus. Instead, several genera express these enzymes in tandem. Encouraging a community‐level approach to bioremediation may therefore lead to more resilient and efficient strategies for managing plastic pollution in marine environments [71]. Additionally, by promoting research on microbial plastic degradation, policymakers can address critical gaps in understanding the ecological roles of these organisms, which could inform environmental risk assessments and the development of new, biodegradable plastic alternatives [21, 31]. Together, these findings underscore the potential of diverse microbial communities within marine plastispheres to contribute to plastic biodegradation, highlighting the importance of these natural processes as a foundation for sustainable plastic waste management solutions.

## 5 Conclusion

To date, the molecular mechanisms underpinning the initial days of microbial colonisation of marine plastic debris have remained uncharacterised. To resolve the interactions between the earliest colonisers and plastic surfaces, we utilised multi‐omics to investigate the activity of the marine plastisphere community grown on LDPE for 3 and 7 days. Our findings reveal a complex relationship between pioneering and secondary colonisers, shaped by stressors such as ROS and nutrient limitation, whilst also highlighting the interplay of cooperation and competition among microorganisms. Consistent with previous research, the majority of these nascent plastisphere microorganisms were primarily composed of heterotrophic bacteria, indicative of marine plastisphere communities found in colder environments. The near‐complete assembly of metagenome‐assembled genomes (MAGs) for *Pseudomonas* and *Marinomonas* significantly enhanced our ability to characterise the associated proteomes and elucidate their precise functions within the dynamic young plastisphere. In this study, *Pseudomonas*, the pioneering genera, was likely aided through its known ability to form biofilms, whilst the genera detected in the later plastisphere diversified over time, potentially fuelled by metabolites produced within these biofilms. Proteins related to the biodegradation of plastic and other xenobiotics were expressed by *Pseudomonas*, *Marinomonas* and three additional genera, highlighting the potential for these microorganisms to contribute to plastic biodegradation processes. Further studies exploring the functioning of the nascent plastisphere are required, to determine their role in those processes. Overall, this study significantly enhances our understanding of the early formation of the plastisphere and its interactions with plastic, providing valuable insights essential for addressing the growing environmental challenge of plastic pollution.

## Conflicts of Interest

The authors declare no conflicts of interest.

## Associated Data

The mass spectrometry proteomics data have been deposited to the ProteomeXchange Consortium (https://proteomecentral.proteomexchange.org/) via the MassIVE partner repository [72, 73] with the dataset identifier PXD056358.

## Supporting information




**Supplementary File_S1** Overview of the total metaproteome identified at D3 and D7 showing the taxonomic annotation (LCA score > 80%) obtained from mPies using our Combined‐Metagenome Database.

**Supplementary File_S2** Protein identification obtained from the publicly available databases for antimicrobial resistance (CARD), plastic metabolism (PlasticsDB), and virulence factor (VFDBs).

**Supplementary File_S3** Proteome Summaries at 1% FDR for Metagenome‐Assembled Genomes (MAGs) of *Pseudomonas* and *Marinomonas*

**Supplementary File_S4** Fold‐change analysis summary using Skyline when comparing protein abundance ratio at D7 and D3.

**Supplementary File_S5** Peptide Summary Exports generated by Protein Pilot, including peptide sequences (Conf ≥50), and associated metadata for each condition and replicate.

**Supplementary File_S6** Protein Summary Exports generated by Protein Pilot, and associated metadata for each condition and replicate.

## Data Availability

The data that support the findings of this study are openly available in ProteomeXchange at https://proteomecentral.proteomexchange.org/, reference number PXD056358.
